# Minimally Invasive Total Hip Arthroplasty in a Patient with Hereditary Multiple Exostoses: A Case Report

**DOI:** 10.5704/MOJ.1811.010

**Published:** 2018-11

**Authors:** A Santoso, P Utomo, CJ Im, KS Park, TR Yoon

**Affiliations:** Department of Orthopaedic and Traumatology, Sebelas Maret University, Solo, Indonesia; *Center for Joint Disease, Chonnam National University Hwasun Hospital, Jeonnam, Republic of Korea

**Keywords:** hereditary multiple exostoses, total hip arthroplasty, minimally invasive surgery

## Abstract

Hip geometry abnormalities found in patients with hereditary multiple exostoses (HME) could promote premature hip joint degeneration which needs treatment. We report the case of a 45-year old male with right hip arthrosis who underwent two-incision minimally invasive (MIS-2) total hip arthroplasty (THA), with satisfactory outcome. This technique could be an alternative approach for performing THA in patients with hereditary multiple exostoses.

## Introduction

Various hip abnormalities occur in patients with hereditary multiple exostoses. They include coxa valga, acetabular dysplasia, joint subluxation and exostosis around the hip joint^1^. These abnormalities could predispose the hip joint to premature degeneration, and may require more extensive surgical exposure due to enlarged femoral head. There are a few recent reports about THA in patients with HME^1-5^. We report a case of hip arthrosis in a patient with HME which was treated with THA using a minimally invasive two-incision approach. Informed consent has been obtained from the patient prior to this publication.

## Case Report

A 45-year old male presented at our clinic with right hip pain of five months duration and the pain had worsened since the last two months with a visual analog scale (VAS) score of 7. The pain was localised to the hip and it occurred especially during movement and weight bearing. There was no history of trauma, alcohol abuse, steroid use, metabolic disorder, and any associated chronic disease. His height was 161cm, body weight was 63kg, and body mass index was 24.3 kg/m^2^. Physical examination revealed a positive Patrick’s test. Range of movement at the right hip joint was flexion 100 degrees, abduction 40 degrees, adduction 10 degrees, external rotation 40 degrees, and internal rotation 10 degrees. Several bony lumps were found at various periarticular sites on the upper and lower extremities. Preoperative functional activity score based on Harris Hip score (HHS) was 40. A plain anteroposterior pelvic radiograph showed right femoral head flattening with subchondral sclerosis and a cyst. Joint space obliteration and joint subluxation were also observed.

**Fig. 1:**
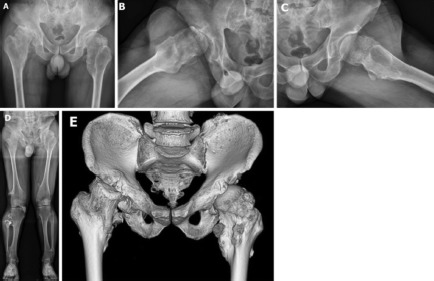
(a) Anteroposterior pelvic radiograph showing femoral head collapse and subluxation, with exostoses present on the inferomedial femoral neck on both sides. (b) Right femoral head lateral radiograph. (c) Left femoral head lateral radiograph. (d) Teleroentgenogram of the lower extremity showing several exostoses in the periarticular region. (e) 3D-scan confirmed the exact position of the proximal femoral exostosis.

Exostoses were found at the inferomedial site of the base of femoral neck on both sides, and on the superolateral side of the left femoral neck (Fig. 1a). Proximal femoral geometrical measurements on both anteroposterior hip radiographs revealed the following findings (right/left): femoral head width 66.8 mm/74 mm, widest femoral neck width 79.5 mm/74.5 mm, bilateral coxa valga with a neck-shaft angle 157 degrees/157 degrees, bilateral acetabular dysplasia with Sharp’s angle 43 degrees/43 degrees, right hip joint subluxation of 48% and left femoral head coverage of 69% without any sign of joint subluxation. Radiographic limb length discrepancy was 17mm. Femoral head width on the lateral femoral head radiograph was 59.6 mm/59.7 mm with femoral neck width 57.1mm/53.6mm (Fig. 1b, 1c). The anteroposterior teleroentgenogram of the lower extremity showed multiple exostoses on both distal femoral, proximal tibial, and distal tibial regions (Fig. 1d). Pelvic computed tomography confirmed the exact position of proximal femoral exostosis (Fig. 1e). Based on the physical and radiographic findings, this patient was diagnosed as having right coxarthrosis associated with HME. The patient was scheduled for THA surgery.

**Fig. 2:**
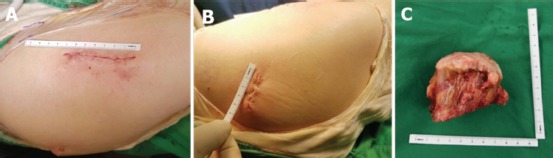
(a) The first anterolateral incision (7 cm) after wound closure. (b) The second posterolateral incision (4 cm) after wound closure. (c) The excised femoral head and neck (size: 7x6x5 cm).

Details of MIS-2 incision THA have been provided in a previous publication^2^. Briefly, the patient was positioned in the lateral decubitus position. The hip joint was approached via two incisions. The first incision was part of Watson-Jones anterolateral approach with muscle interval between the gluteus medius and tensor fascia lata. This incision started from a point approximately one finger breadth posterior to the anterior border of the greater trochanter, just distal to the vastus ridge, and extended cranially and anteriorly at an angle of 30 degrees to the long axis of the femoral shaft. The length of the incision was 7cm (Fig. 2a). Through this incision, femoral head extraction was performed (Fig. 2c), acetabular reaming and insertion of the acetabular cup. Excision of proximal femoral exostosis was also performed through the anterolateral incision. The second incision of 4cm length (Fig. 2b) was performed through part of the posterolateral incision with the hip flexed to 90 degrees. After dissecting through the muscle fibers of gluteus maximus and fat excision, the piriformis tendon was exposed and used as a landmark. Intermuscular dissection with interval between gluteus medius and piriformis muscle was performed to expose and incise the posterosuperior capsule. Femoral canal broaching and stem position was confirmed by fluoroscopy. The femur was then brought anteriorly by applying traction, external rotation and extension of the hip. The femoral head trial and head implant were inserted through the anterolateral incision after fluoroscopic confirmation of leg length discrepancy. The femoral component was cementless Master-SL® [Lima Corporate, Udine, Italy]. The acetabular component was cementless Delta-PF ® cup [Lima Corporate, Udine, Italy] with a diameter of 50mm. Ceramic-on-ceramic bearing was used with a head diameter of 36mm. The posterior and anterior joint capsule, fascia lata, and subcutaneous tissue were repaired; and the skin closed. The total skin-to-skin operation time was 55 minutes.

**Fig. 3:**
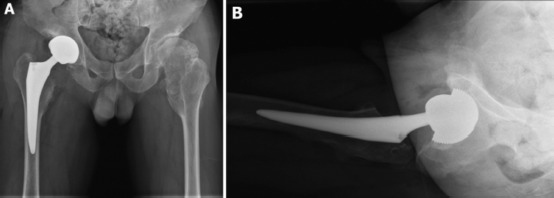
(a) Postoperative anteroposterior pelvic radiograph showing no limb length discrepancy. (b) The right lateral hip radiograph showing proper acetabular component placement.

A postoperative hip radiograph showed satisfactory femoral and acetabular component placement with an equal limb length compared to the contralateral side (Fig. 3). Postoperative protocol which included an abduction pillow between the two legs and elastic stockings to prevent deep vein thrombosis were applied. Early hip range of motion exercise was allowed on the first day after surgery. Walking with tolerable weight bearing with crutches was started on the second postoperative day. Range of movement at the right hip joint had improved: flexion 120 degrees, abduction 40 degrees, adduction 30 degrees, external rotation 40 degrees, and internal rotation 30 degrees. No complication occurred after surgery. At two years follow-up, the patient had an excellent hip function with a HHS of 96.

## Discussion

Abnormal geometry in the hip joint of patients with HME has made THA a challenging procedure. A previous study by Porter *et al* reported that the hip joint of patients with HME has a greater neck-shaft ratio compared to that in the normal population. This occurred due to overgrowth of the femoral neck^1^. Furthermore, the presence of exostosis around the hip joint resulted in an even larger bone. In this present case, exostoses were present on both medial sides of the base of the femoral neck. As a result, the base of the right femoral neck had an approximate width of 8cm. The possible consequence of the relatively larger proximal femoral geometry is more lax periarticular soft tissue structures (capsule and muscle), which laxity, unfortunately, will be increased after THA. Vaishya *et al* reported that bilateral THA was performed in patients with HME using an anterolateral approach. They reported the occurrence of prosthesis dislocation at about one week after surgery which needed revision arthroplasty surgery to prevent a further episode of dislocation^3^.

The other consequence of a larger femoral geometry was the possibility of iatrogenic greater trochanteric fracture during a surgical dislocation maneuver in THA surgery as reported by Kanda *et al*^4^. Hence, other authors preferred to use the extensile approach with extended trochanteric osteotomy for performing THA in patients with HME^5^. However, we believed that the risk of postoperative complication will possibly be increased with this kind of approach.

In this present case, we performed a MIS-2 incision THA. With this approach, we used the inter-muscular space to reach the joint. No important muscles including the hip external rotators and the gluteus medius muscle were detached during the procedure. While closing the wound, it was also possible to perform joint capsule repair and tightening because we only made a joint capsule incision (no excision) during our surgical procedure. We believed that this would reduce the possibility of postoperative prosthesis dislocation due to soft tissue laxity. Dislocation maneuver was not needed prior to femoral neck osteotomy. It was also possible to perform the removal of exostosis through the first anterolateral incision.

The other important factor to consider was the presence of coxa valga. We found that the NSA in our patient was 157 degrees on both sides. The presence of coxa valga is very important with respect to implant selection and placement during adjustment of the patient’s leg length especially for THA in a unilateral case. In this present case, we used a standard offset femoral stem under fluoroscopic guidance during adjustment of the patient’s leg length intraoperatively. The use of fluoroscopy was also very important when performing THA with this MIS-2 incision approach, especially during preparation (broaching) of femoral canal and stem insertion as it is performed through a very small posterior incision with no direct visualisation. Care has to be taken to avoid intraoperative periprosthetic femoral fracture.

We suggest that two-incision minimally invasive total hip arthroplasty should be considered for patients with hereditary multiple exostosis in view of less soft tissue injury and potentially lower risk of post-operative instability. Bigger cohort study will be necessary to provide stronger evidence to support this indication.

## Conflict of Interest

The authors declare no conflicts of interest.
